# Research Progress on the Preparation and Function of Antioxidant Peptides from Walnuts

**DOI:** 10.3390/ijms241914853

**Published:** 2023-10-03

**Authors:** Yuxi Hu, Ce Ni, Yingying Wang, Xun Yu, Hao Wu, Jia Tu, Changzhu Li, Zhihong Xiao, Li Wen

**Affiliations:** 1 School of Food Science and Bioengineering, Changsha University of Science & Technology, Changsha 410114, Chinahaowu@csust.edu.cn (H.W.); 2 State Key Laboratory of Utilization of Woody Oil Resource, Hunan Academy of Forestry, Changsha 410004, China; 3 School of Food and Biological Engineering, Hefei University of Technology, Hefei 230009, China

**Keywords:** walnuts, antioxidant peptides, oxidative stress, structure–activity relationship

## Abstract

Food-derived peptides have good antioxidant activity and are highly safe for humans; consequently, there has been continuous growth in research on antioxidants, with potential applications in food, medicine, cosmetics, and other fields. Among food-derived peptides, walnut-derived peptides have attracted increasing attention as food-derived peptides rich in eight essential amino acids. This review summarizes the progress made in the development and identification of antioxidant peptides in walnut proteins. This article mainly describes the interaction between reactive oxygen species and cellular antioxidant products, modulation of enzyme content and activity, and regulation of the redox signaling pathways and analyzes the mechanisms of reduction in oxidative stress. Finally, the complex structure–activity relationships of walnut-derived peptides are analyzed based on their amino acid composition and secondary structure of the polypeptides. This review provides a theoretical basis for the production of walnut-derived antioxidant peptides and could help promote the development of the walnut industry.

## 1. Introduction

Biologically active peptides (BAPs) are composed of amino acids generated by proteolysis, and their different compositions and arrangements result in different physiological functions. Currently, BAPs are reported to possess various functional properties such as antimicrobial, anticholesterol, antioxidant, antiallergic, antithrombotic, anticancer, antidiabetic, and immunomodulatory properties [1,2]. In recent years, research on BAPs from plants has received increasing attention because of the advantages of abundant raw material variety and low cost. The active peptides isolated from plants such as walnuts, peas, and rice have been scientifically proven to possess many beneficial effects [3,4,5].

**Figure 1:**
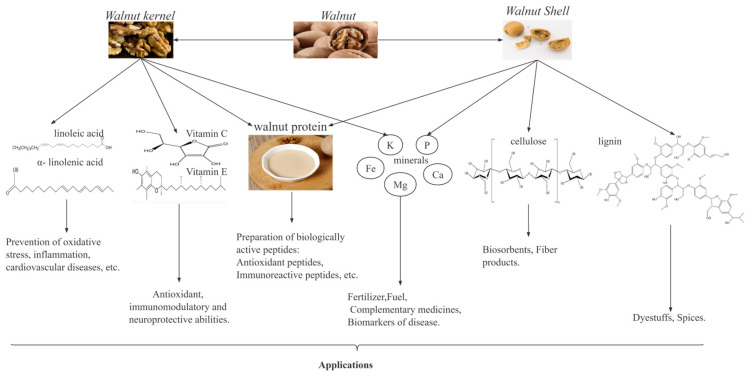
Composition of walnuts and their uses.

Walnuts, as a traditional plant, are one of the most important economic forest products in the world, particularly in China. According to the FAO data, from 2017 to 2021, the global walnut harvest area and yield steadily increased, with year-on-year growth rates of 2.87% and 1.59%, respectively. In 2021, the walnut harvest area and yield of China accounted for 24.6% and 31.42% of the world’s total, respectively, thus making China the leading country in global walnut production. As shown in Figure 1, walnut kernels are rich in unsaturated fatty acids, proteins, vitamins, and minerals [6,7], which have important biological activities such as antioxidant [8], antitumor [9], anti-inflammatory [10,11], anti-hypertensive [12] and anti-cardiovascular disease activities. Walnut kernels have a high fat content (52–70%), and these fats can be processed into walnut oil [13]. Walnut oil is rich in unsaturated fatty acids and is favored by people because of its nutritional benefits [14]. In recent years, the increase in market demand for walnut oil has led to an increase in walnut residue, a byproduct of walnut oil. Walnut residue contains approximately 40–45% proteins, which are rich in 18 amino acids, including the eight essential amino acids for the human body, thus making walnut residue a high-quality plant protein resource [13,15]. Therefore, research on walnut proteins is valuable and potentially useful for developing functional products with health benefits.

Free radicals are the inevitable products of metabolic processes in the human body; these radicals cause certain damage to the human body, such as acceleration of the aging process and induction of cardiovascular diseases [16]. Antioxidant peptides can reduce the peroxidation reaction in the human body and neutralize free radicals.

Antioxidant peptides are classified as natural antioxidant peptides and synthetic antioxidant peptides [17]. Synthetic antioxidants have many safety issues when used in excess, such as teratogenic and carcinogenic effects; consequently, with the increasing demand for food safety and more awareness of the health risk posed by synthetic antioxidants, people have focused their attention to natural antioxidants [18,19]. Natural antioxidant peptides are a type of peptide that can be processed and extracted from proteins of living organisms; these peptides have a high safety profile and strong antioxidant activity [20].

Food-derived antioxidant peptides are peptides with antioxidant activities, and they are obtained by enzymatic digestion of edible proteins [21]. In recent years, food-derived antioxidant peptides have become a research hotspot because of their low molecular weight (MW), simple structure, easy absorption by the human body, high antioxidant activity, and strong stability under different environmental conditions [18]. The antioxidant peptides generated by the enzymatic digestion of walnut proteins have positive effects on learning and memory and have high potential for application in synergistic anticancer and sleep improvement therapies; in particular, the neuroprotective effects of walnut antioxidant peptides have been widely reported [15,22,23].

In the present review, the preparation, isolation, purification, and identification methods of walnut antioxidant peptides are summarized, and the evaluation methods, mechanisms of action, and constitutive relationships of antioxidant peptides are discussed. Furthermore, the development of other food-derived antioxidant peptides is discussed and compared with walnut antioxidant peptides. This review also highlights the gaps in the research and use of walnut antioxidant peptides and suggests the potential impact of these peptides on human health and their application in the food and pharmaceutical industries.

## 2. Preparation, Separation, Purification, and Identification of Antioxidant Peptides

### 2.1. Preparation of Antioxidant Peptides

Currently, various methods are used to prepare antioxidant peptides from food sources, including microbial fermentation, enzymatic hydrolysis, gene recombination, chemical synthesis, and new methods, such as ultrasonic-assisted hydrolysis, pulsed electric field (PEF), and microwave-assisted extraction.

#### 2.1.1. Chemical Extraction

Chemical extraction of polypeptides is a well-established and conventional preparation method. Some studies have explored traditional and unconventional methods for protein extraction from Irish brown algae.

In one study, cannabis seed protein hydrolysate prepared from defatted cannabis seeds by using alkaline lysis/acid precipitation was used. Cannabis seed protein hydrolysate was further isolated and characterized to obtain four peptides with good angiotensin-converting enzyme (ACE) inhibitory IC_50_ values of 16 ± 1.5, 145 ± 13, and 526 ± 33 µM for GVLY, LGV, and RVR, respectively [24].

However, chemical extraction has disadvantages of large consumption of chemical reagents, prolonged completion time, high environmental pollution, and high equipment cost [25]. Moreover, although chemical hydrolysis produces a large number of small molecule peptides, during the hydrolysis process, high concentrations of byproducts are generated, resulting in low peptide recovery rates [26]. Therefore, chemical extraction is not currently the preferred method for preparing food-derived antioxidant peptides.

#### 2.1.2. Microbial Fermentation

Microbial fermentation is a biotechnological process for obtaining bioactive peptides. The process involves the use of microorganisms capable of producing hydrolytic enzymes that can hydrolyze proteins into shorter peptides; usually, these microorganisms include bacteria, fungi, or yeast, which are indigenously present in the substrate or externally added as fermenting agents [27,28]. Microbial fermentation can hydrolyze proteins and produce enzymes, and the resulting enzymes can also hydrolyze proteins, thus reducing the cost of hydrolysis [29].

In a study by Wu et al. [22], optimizing the fermentation conditions of *Bacillus subtilis* by the response surface method (RSM) yielded a walnut peptide with a high antioxidant capacity. This antioxidant peptide showed better DPPH radical scavenging capacity and Fe^2+^ chelating activity than glutathione (GSH) at the same concentration.

Because each microorganism (bacteria, fungus, or yeast) has a different proteolytic system, the fermentation of each microorganism produces different hydrolytic enzymes [30]. The microbial fermentation process is also sensitive to the environmental conditions, and small changes in pH, temperature, and pressure during the process can produce undesirable results, leading to the unstable quality of the final product; moreover, prolonging the fermentation time can lead to safety issues such as microbial infections [28,31]. Therefore, this approach requires a careful selection of microorganisms and their fermentation conditions to avoid safety issues.

#### 2.1.3. Enzymatic Hydrolysis

In contrast, enzymatic hydrolysis has the advantages of mild conditions, easy control, higher specificity, and fewer byproducts; hence, enzymatic hydrolysis has become the primary method to prepare antioxidant peptides [25].

Enzymatic hydrolysis requires high specificity of enzymes. As shown in Table 1, the enzymes commonly used for enzymatic hydrolysis include trypsin [32], pepsin [33], alkaline protease [34], flavor protease [8], and papain [35]. Among these enzymes, alkaline protease exhibits a higher enzymatic efficiency in preparing bioactive peptides from walnuts.

As shown in Table 1, alkaline protease is commonly used for walnut protein hydrolysis. Some studies have shown that calcineurin was effective in isolating a new antioxidant peptide from pecan protein isolate (PPI) obtained from hickory, with the amino acid sequence LAYLQYTDFETR. Overall, the peptide at a concentration of 0.1 mg/mL exhibited 67.67%, 56.25%, and 47.42% ABTS, DPPH, and hydroxyl radical scavenging activity, respectively. This peptide has good application prospects in functional foods and in preventing oxidative reactions during food processing [36]. Similarly, Zhang et al. showed that the DPPH and hydroxyl radical scavenging capacity of protein hydrolysates hydrolyzed by alkaline protease exceeds 60%, and the obtained hydrolysates show significantly higher antioxidant activity than the original protein [37].

Enzymatic hydrolysis has several drawbacks, including long enzymatic digestion time, low efficiency, and potential impact of enzymes on the purity of peptides. However, it also has advantages such as mild conditions, easy control of the process, and high specificity. Thus, it remains the frequently used method to prepare antioxidant peptides.

#### 2.1.4. Chemical Synthesis

Chemical synthesis of antioxidant peptides is mainly achieved by condensation of amino acids [38]. For synthetic raw materials that contain amino acid monomers with more than two functionalities, the groups that do not need to react are temporarily protected to obtain synthetic polypeptides with specific sequences, and linkage reactions are then performed to ensure the directionality of the synthesis [34].

For walnut antioxidant peptides, this approach has been commonly used in recent antioxidant mechanism studies to precisely elucidate the relationship between walnut peptides and antioxidant functions. One study identified and screened four walnut peptides with anti-inflammatory activity by using the solid-phase peptide synthesis method for chemical synthesis. The anti-inflammatory effect of these four peptides on lipopolysaccharide (LPS)-induced RAW 264.7 cells were studied, and the peptide with the strongest anti-inflammatory activity was confirmed [30]. In another study, a peptide (EVSGPGLSPN) with strong antioxidant properties was obtained by purification, isolation, and identification. Specifically, the peptide was chemically synthesized by a solid-phase peptide synthesis method to investigate its potential protective mechanism against hydrogen peroxide-induced neurotoxicity in PC12 cells [39].

The generation of synthetic peptides requires expensive solid-phase materials and excessive reagents, substrates, and solvents, leading to a high production cost [40]. Hence, it is crucial to develop synthetic methods that are highly productive, cost effective, and less wasteful.

#### 2.1.5. Gene Recombination

Gene recombination is frequently used to synthesize peptides after purification and identification; this approach can be used to replace, add, or delete any part of the amino acid residues in a peptide. By using this technique, the structural and functional relationships of peptides can be elucidated more precisely; moreover, a variety of novel artificial peptides can be created for studying antioxidant peptides [41,42].

Wang et al. [43] chemically synthesized the DNA coding sequence of GVYPHK and tandemly formed 10 copies of the sequence. The synthesized sequence was cloned into the vector pET-15b and expressed in *Escherichia coli* BL21 (DE3) cells. The experimental results showed that a single oral dose of this peptide in spontaneously hypertensive rats significantly reduced systolic blood pressure at 2 h after administration [43].

As an emerging peptide synthesis method, gene recombination is currently limited to medical applications because of its technical complexity and has not been widely applied in the field of food-derived peptides. The utilization of the gene recombination method is being considered as an important direction for future research on walnut peptides.

#### 2.1.6. Other Methods

In recent years, ultrasound-assisted hydrolysis, microwave-assisted extraction, and PEF treatment [44,45,46] have emerged as new processing techniques for the production of bioactive peptides through enzymatic hydrolysis. The data indicate that PEF treatment improves the ability of peptides to scavenge free radicals, thereby enhancing their protective capability to inhibit oxidative stress and scavenge reactive oxygen species (ROS) [44].

Compared with the other techniques for obtaining food-derived peptides, these methods have been used by relatively few studies in the field of walnut peptide hydrolysis. In the study of Wang et al. [47], ultrasound-assisted pretreatment improved the enzymatic hydrolysis of walnut proteins, with an increase in the extraction rate by approximately 10% as compared with that of the control group. Moreover, because of a synergistic effect between ultrasound and enzymatic digestion, ultrasound-assisted hydrolysis can improve the enzymatic digestion level and increase the extraction rate of walnut proteins [48].

Because these methods are less frequently used for producing walnut antioxidant peptides, particularly the PEF treatment method with microwave assistance, future studies could focus on analyzing the potential of these methods for improved application in this field.

### 2.2. Purification, Isolation, and Identification of Walnut Antioxidant Peptides

Walnut protein hydrolysate (WPH) is a complex mixture of partially unhydrolyzed proteins; peptides with diverse chain length, hydrophobicity, and net charge; and free amino acids. To more accurately assess the structural and functional characteristics of antioxidant peptides in WPH, it is critical to develop suitable purification methods [33]. In recent years, several novel separation techniques have emerged, including column chromatography, high-performance liquid chromatography (HPLC), reverse-phase HPLC, ultrafiltration membrane separation (ultrafiltration and nanofiltration), gel permeation chromatography, and various combinations of these techniques [49].

**Figure 2:**
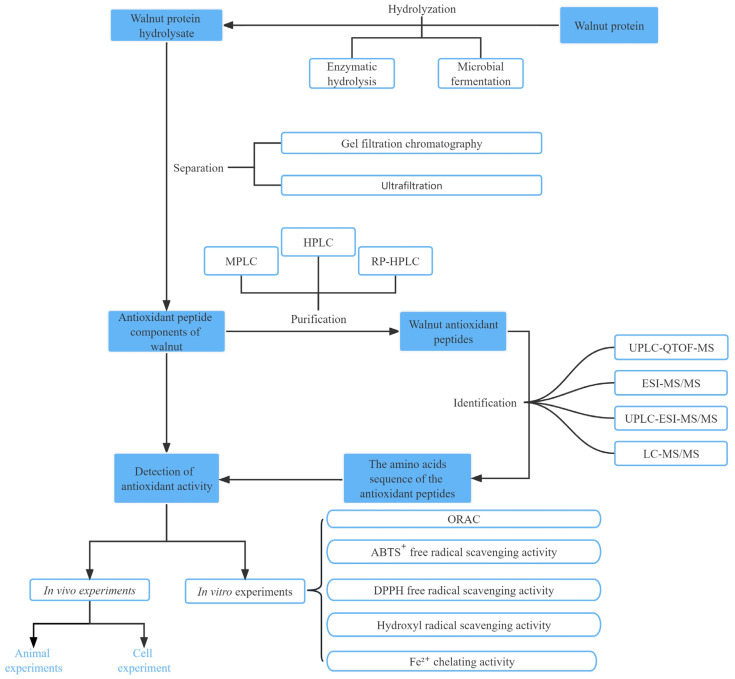
Schematic diagram of the preparation process and function analyses of walnut antioxidant peptides.

Table 1 summarizes some of the methods used for the purification, isolation, and identification of walnut antioxidant peptides. Among these methods, ultrafiltration membranes are typically used for the preliminary extraction of antioxidant peptides, leading to the isolation of peptides with varying MWs. Column chromatography alone is relatively weak in separating proteins and is frequently used in conjunction with HPLC. HPLC offers excellent separation efficacy, sensitivity, sample capacity, and recovery. However, because of the high cost and time-consuming process, HPLC is best employed in combination with other separation techniques to achieve superior separation and purification [50]. Crude separation of peptides using an ultrafiltration membrane allows to separate proteolytic products into peptides with different MWs. In one study, walnut peptides with MWs below 3 kDa were collected using ultrafiltration membranes for ACE inhibitory peptide studies; the ACE inhibitory activity of these peptides was 88.6% at 1 mg/mL concentration, which was higher than that of hydrolyzed proteins not subjected to ultrafiltration at the same level (47.3%) [51]. This method has high potential for application because of its simple operation, high separation efficiency, and ease of industrial scale-up [52]. These separation and purification techniques are widely implemented for isolating and purifying walnut peptides, with the combination of various separation methods being considered a crucial approach to isolate highly active antioxidant peptides in contemporary studies (Figure 2).

Hu et al. conducted ultrafiltration-based separation of the hydrolysis products of PPI by using membranes with cutoff MWs of 10, 5, and 3 kDa [36]. The antioxidant activities of the resulting fractions were evaluated in vitro, and the most potent fraction (<3 kDa) was successfully purified to identify novel antioxidant peptides. Studies have shown that highly active walnut peptides generally contain fewer than 20 amino acids and belong to the low MW range. Feng et al. [33] separated the defatted WPH into five fractions with different MWs by using ultrafiltration membranes and further separated and purified two new fractions by gel filtration chromatography. Fractions with the highest antioxidant activity were obtained by purification with reverse-phase HPLC.

After isolating and purifying antioxidant peptides, their structures need to be determined. The commonly used methods to identify peptides include DNA translation, mass spectrometry (MS), and peptide sequencers. MS is widely used for amino acid sequence identification because of its high efficiency, sensitivity, and reproducibility [25]. Tandem MS (MS/MS), MS, liquid chromatography–tandem MS (LC-MS/MS), ultra-performance liquid chromatography (UPLC), and quadruple time-of-flight MS (Q-TOF-MS) are currently used for peptide identification. Depending on the different working principles of mass spectrometers, the types of mass spectrometers mainly include fast atom bombardment, electrospray ionization (ESI), and matrix-assisted laser desorption ionization time of flight (MALDI-TOF). ESI and MALDI-TOF MS are commonly used to identify antioxidant peptides because of high sensitivity and low detection limits. The mass-to-charge ratio (*m*/*z*) of the peptide subfractions is obtained using MS and then entered into an online protein database to determine the amino acid sequence. If the online protein database does not contain primary structural information of the parental protein, a peptide sequencer can be used for de novo sequencing of the peptide sequence [1,16]. However, this peptide sequencer is unsuitable for analyzing peptide samples with low purity [53]. Liquid chromatography (LC) combined with tandem MS is also widely used to analyze peptide sequences, including LC-ESI-MS/MS, LC-MALDI-TOF-MS/MS, LC-Q-TOF-MS/MS, and LC-FTICR (Fourier transform ion cyclotron resonance)-MS/MS.

## 3. Activity Analysis and Functional Study of Antioxidant Peptides

Presently, the antioxidant activity of walnut peptides is detected through in vivo and in vitro methods. The in vitro antioxidant activity is generally evaluated based on three aspects: evaluation of anti-lipid peroxidation ability, determination of free radical scavenging ability, and assessment of reducing power or metal ion chelating ability [54]. The in vitro antioxidant activity evaluation method can simply and rapidly determine whether polypeptides have antioxidant activity, and it is frequently used in preliminary studies to characterize the antioxidant activity of target peptides [55]. Table 1 lists the in vitro methods used to evaluate the antioxidant capacity of some walnut peptides.

In recent years, antioxidant activity assays at the cellular and animal level have become the primary method to assess the antioxidant capacity of peptides. Because animal models are expensive and have low generalizability, cellular models are considered a more valuable tool due to their rapidity and low cost. Therefore, cellular models are a better choice than animal models and human clinical trials for studying antioxidant activity. Compared with in vitro antioxidant activity assays, cellular assays showing the cytoprotective effects of antioxidant peptides on damaged cells provide more valuable insights into the mechanisms through which antioxidant peptides exert protective effects against oxidative stress. Cellular models also provide biologically relevant information such as cellular uptake, distribution, and metabolism of antioxidant compounds. To conclude, cellular models are a preferred method to assess the antioxidant activity of walnut antioxidant peptides because of their cost effectiveness, rapidity, and biological relevance [56].

In general, the antioxidant activity of peptides is attributed to their diverse abilities, mainly including regulation of ROS and intracellular antioxidant activity, modulation of enzyme content and activity, and interactions with the redox signaling pathways.

### 3.1. Regulation of ROS

Oxidative damage is caused by excessive free radicals, leading to cell damage or death, thereby triggering the development of diseases such as cancer, neurodegenerative diseases, diabetes, and other diseases [57]. During the process of oxidative stress, a large amount of ROS is produced. ROS can damage biological macromolecules such as DNA, proteins, and fatty acids [58]. Walnut antioxidant peptides can capture or neutralize free radicals by delaying oxidation and production of free radicals or by donating hydrogen or electrons, thereby making free radicals inactive and preventing or terminating their chain reactions. ROS include free radicals such as superoxide anion (O^2−^), hydroxyl radical (HO), alkoxyl radical (RO), and alkoxy peroxyl radical (ROO) as well as non-free radical substances such as singlet oxygen (1O_2)_, hypochlorous acid (HOCl), ozone (O_3_), hydrogen peroxide (H_2_O_2_), and peroxynitrite (ONOO^−^). Antioxidant peptides can directly eliminate ROS by donating hydrogen or electrons [59]. Many methods are available to evaluate the antioxidant activity of walnut peptides through ROS regulation. Currently, the most widely used in vitro evaluation methods for walnut peptides in China and abroad include DPPH free radical scavenging activity assay, ABTS+ free radical scavenging activity assay, oxygen free radical scavenging ability assay, and hydroxyl free radical scavenging activity assay.

Ren et al. [60] evaluated the effects of Manchurian walnut hydrolyzed peptide (MWHP) on ROS and glutathione peroxidase (GSH-Px) production in H_2_O_2_-induced PC12 cells. The results showed that compared with the positive control group (H_2_O_2_ injury group), the three MWHP groups, particularly the <3 kDa MWHP group, inhibited ROS production. Jahanbani et al. also used ROS to assess the effect of WPH on human breast (MDA-MB231) and colon (HT-29) cancer cell lines. The results showed that walnut antioxidant peptides exert antioxidant effects on these cells. The authors concluded that the antioxidant and anticancer activities of WPHs were significantly related [61].

### 3.2. Regulation of Endogenous Antioxidant Enzyme Activity

Antioxidant peptides not only reduce ROS production but also enhance the defense capacity of enzymes and nonenzymatic antioxidants. The main antioxidant enzymes include superoxide dismutase (SOD), catalase (CAT), and GSH-Px. These enzymes can eliminate excess ROS and protect brain cells from oxidative damage. Among them, SOD catalyzes the dismutation of superoxide to produce H_2_O_2_ and O_2_, CAT catalyzes the decomposition of H_2_O_2_ to produce H_2_O and O_2_, and GSH-Px protects cell membranes from lipid peroxidation damage [44]. Biomarkers are the products of lipid peroxidation in the cell membrane, and their production can exacerbate membrane damage. The correlation between enzyme activity and biomarker production can be elucidated based on the observation that increased levels and functions of internalized antioxidants can hinder the production of biomarkers. Therefore, biomarkers are also commonly used to evaluate the antioxidant capacity of bioactive peptides [18].

Biomarkers mainly include malondialdehyde (MDA), low-density lipoprotein (LDL), and 8-hydroxy-2′-deoxyguanosine (8-OHdG). MDA is a secondary end product of lipid peroxidation; hence, the reduction of intracellular MDA levels in conjunction with the increase in antioxidant enzyme activity is frequently used to evaluate the antioxidant ability of peptides [62]. A previous study showed that the walnut globulin antioxidant peptides dose dependently increased SOD and CAT levels in mice while reducing MDA levels, thereby significantly improving the antioxidant capacity of mice [63]. Jia et al. [64] established a D-galactose-induced oxidative damage model in Sprague Dawley (SD) rats to study the antioxidant effects of walnut peptides. The results showed that high-dose walnut peptides (WPH) increased the activity of SOD and GSH-Px in the liver and serum of rats and decreased MDA levels; the effect was better than that of the VE control group, indicating that walnut peptides have good antioxidant activity.

Caspase is a family of proteases that play an important role in the process of apoptosis and is also commonly used in studying antioxidant peptides. Liu et al. [35] explored the mechanism through which walnut antioxidant peptides exert neuroprotective effects on zebrafish by measuring the inhibitory effects of caspases 3/7, 8, and 9. The results showed that walnut antioxidant peptides at a moderate dose inhibited the activity of caspases 3/7 and 8, thus demonstrating that walnut antioxidant peptides are a good neuroprotective agent. Liu Dandan [65] induced oxidative damage in SD rats by using D-galactose and studied the in vivo antioxidant effects of walnut peptides. The results showed that walnut peptides significantly reduced the elevated MDA content, decreased GSH content, and reduced total antioxidant capacity due to oxidative damage in the serum, thus indicating good antioxidant activity of walnut peptides.

Currently, although some studies have evaluated the antioxidant activity of walnut peptides by using animal and cell models, most of these studies have focused on the characteristics of antioxidant peptides, such as MW, amino acid composition, primary structure, and secondary structure, rather than the mechanism of their antioxidant activity.

### 3.3. Regulation of Antioxidant-Related Signaling Pathways

The ability of antioxidant peptides to activate endogenous antioxidant damage-related signaling pathways, such as the Keap1-Nrf2-ARE signaling pathway, the Nrf2/HO-1 signaling pathway, and the Akt/mTOR signaling pathway, is crucial in preventing oxidative stress damage in cell tissues [25]. These pathways have shown different antioxidant effects in various cell models. Recent studies have focused on the extraction of food-derived peptides to inhibit these pathways and achieve the desired antioxidant effects [25].

#### 3.3.1. The Akt/mTOR Signaling Pathway

**Figure 3:**
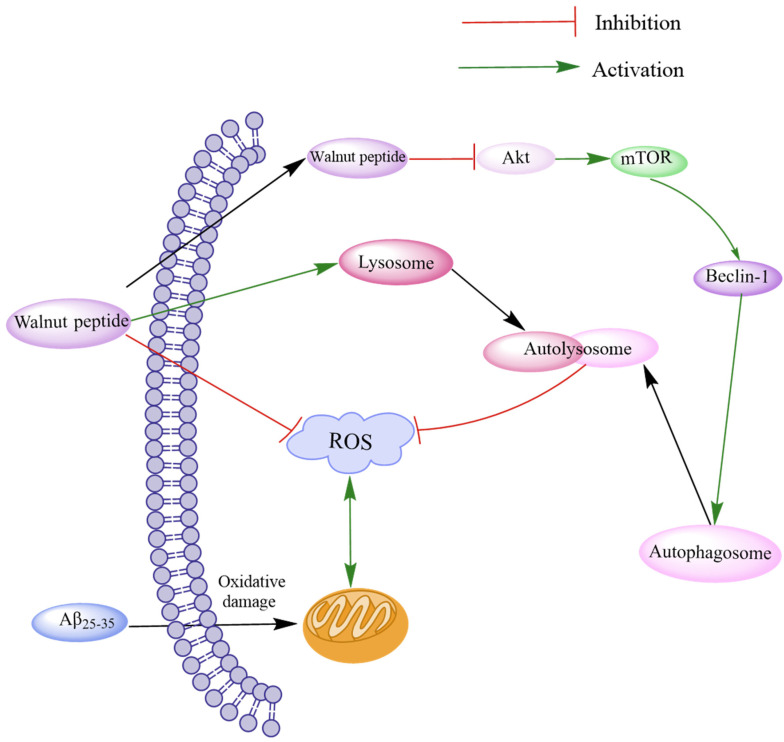
Schematic diagram of walnut peptide regulating autophagy through the Akt/mTOR signaling pathway. The walnut peptide regulates Akt/mTOR signaling pathway by increasing the p-Akt level and reducing the p-mTOR level, thus increasing the expression levels of LC3-II/LC3-I and Beclin-1 and decreasing p62 expression level; this reduces neuronal apoptosis and triggers neuronal autophagy. In addition, these peptides promote fusion with lysosomes to form autophagic lysosomes and accelerate ROS removal by increasing the levels of LAMP1, LAMP2, and cathepsin D.

Oxidative stress often leads to Aβ accumulation by inducing ROS production and mitochondrial morphological and functional damage, eventually leading to brain neurotoxicity. Autophagy can suppress neurotoxicity by inhibiting ROS production [66]. The Akt/mTOR signaling pathway, a typical autophagy regulatory pathway, can modulate the expression of LC3-I and p62 proteins by regulating PTEN, p-Akt, and p-mTOR and attenuating the activity of MDA and SOD [67]. Zhao et al. investigated the novel peptides TWLPLPR, YVLLPSPK, and KVPPLLY that inhibit ROS production [64]. Western blotting assay and immunofluorescence assay revealed that these peptides could regulate the Akt/mTOR signaling pathway through p-Akt (Ser473) and p-mTOR (S2481), thereby reducing LC3-II/LC3-I and Beclin-1 levels by increasing p62 expression to promote autophagy. These peptides increased the levels of LAMP1, LAMP2, and histone D and promoted fusion with lysosomes to form autophagic lysosomes, thereby accelerating ROS removal. The results demonstrate that walnut-derived peptides regulate oxidative stress through autophagy mediated by the Akt/mTOR signaling pathway (Figure 3).

#### 3.3.2. The Keap1-Nrf2-ARE Pathway

The Nrf2 transcription factor is a potential neurological therapeutic target [68,69]. In research studies on walnut antioxidant peptides, it is often used along with Keap1 and OH-1 [70,71,72,73].

**Figure 4:**
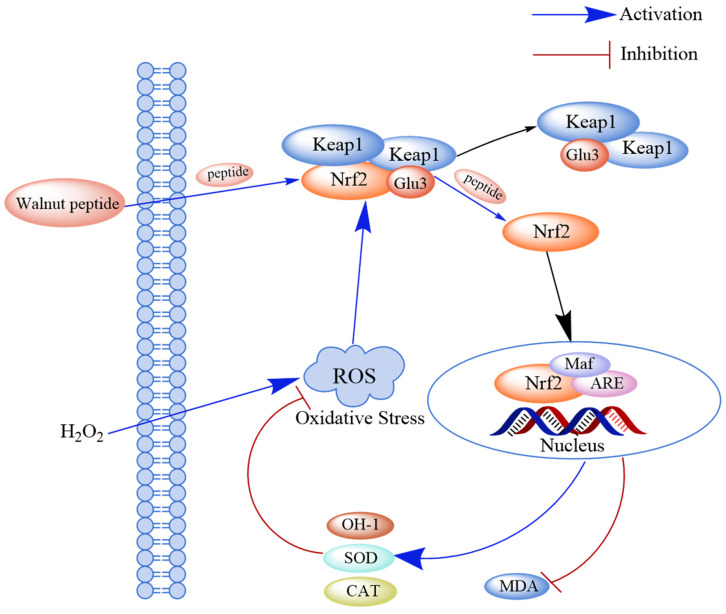
Schematic diagram of the mechanism through which walnut peptide regulates the Keap1-Nrf2-ARE signaling pathway. Peptides directly bind to Keap1 and release Nrf2, thereby activating the Keap1-Nrf2-ARE pathway and inhibiting the formation of the Keap1-Nrf2 complex. Simultaneously, the activity of the antioxidant markers (SOD and CAT) increases in hydrogen peroxide-induced cells.

The Keap1-Nrf2-ARE pathway is a key signaling pathway for endogenous antioxidant damage. Under normal physiological conditions, Nrf2 and its cytoplasmic repressor protein Keap1 are bound in the cytoplasm. However, after cells undergo oxidative stress damage, Nrf2 and Keap1 are separated, and the activated Nrf2 enters the nucleus and binds to its specific chaperone fibrous sarcoma protein (Maf) to form a dimer. The Nrf2-Maf dimer binds to the antioxidant response element (ARE), which regulates the transcription of downstream genes such as phase II detoxification enzymes, antioxidant enzymes, and cytoprotein genes. The expression of these downstream genes can avoid alterations in cellular functions and enhance cellular resistance to oxidative stress. Studies have shown that walnut-derived peptides can inhibit the Keap1-Nrf2-ARE pathway by activating the binding of these peptides to Keap1 and releasing Nrf2 [70] (Figure 4).

#### 3.3.3. The Nrf2/HO-1 Pathway

The Nrf2/HO-1 pathway plays a crucial role in the antioxidation effect by regulating the expression of various anti-inflammatory and antioxidant factors and serving as a central player in the physiological injury response. HO-1, a classical antioxidant enzyme downstream of Nrf2 pathway activation, exhibits potent anti-inflammatory and oxidative stress inhibitory effects [74]. In a study of Gao et al. [71], treatment with the walnut antioxidant peptide WEKPPVSH significantly restored the protein expression of Nrf2 (*p* < 0.05) and HO-1 (*p* < 0.01) and activated the Nrf2/HO-1 signaling pathway. The results suggest that WEKPPVSH may protect LPS-stimulated BV-2 microglia cells from oxidative stress-induced damage by activating the Nrf2/HO-1 signaling pathway. Similarly, Wang et al. investigated the effects of three novel peptides from *Juglans mandshurica* on oxidative stress in HepG2 cells [73]. The results demonstrated that the walnut peptides enhanced nuclear translocation of Nrf2 and HO-1 protein expression, thus protecting HepG2 cells from oxidative stress-induced damage by activating the Nrf2/HO-1 signaling pathway.

There is, however, a lack of studies on walnut antioxidant peptide-mediated regulation of related signaling pathways at the cellular level, such as the analysis of transcription factor expression during the action of the peptides, gene-level study on the signaling pathways during peptide-mediated regulation, and assessment of the cross-talk between walnut antioxidant peptides with various cellular pathways.

## 4. Structure–Activity Relationship Study of Walnut Antioxidant Peptides

Structure–activity relationship (SAR) study has emerged as a valuable tool to predict the activity of bioactive peptides, including ACE-inhibiting, immunoreactive, and antioxidant peptides. Specifically, the antioxidant potential of peptides isolated from proteins is frequently influenced by distinct structural characteristics such as MW, amino acid composition, sequence, and hydrophobicity.

### 4.1. Molecular Weight

Peptides comprising 2–20 amino acids possess stronger antioxidant effects and other bioactive properties as compared with their parent proteins or peptides, because of their enhanced ability to interact with ROS and terminate free radical chain reactions [75]. Moreover, walnut-derived antioxidant peptides with lower MWs have greater potential to penetrate the intestinal barrier and exert antioxidant effects. Previous walnut peptide studies have shown that peptides with MWs less than 3 kDa exhibit the highest antioxidant activity [76].

As shown in Table 1, the antioxidant peptides are primarily composed of 2–6 amino acids, with MWs below 1 kDa. Chen et al. [30] divided the WPH into two fractions, namely WPPH-I (MW > 3 kDa) and WPPH-II (MW < 3 kDa), by using ultrafiltration membranes with a MW cutoff (MWCO) of 3 kDa. The authors demonstrated that the protein hydrolysates with MWs < 3 kDa had greater antioxidant activity than the other fractions. Fei et al. [36] fractionated the WPHs by sequentially using ultrafiltration membranes with MWCOs of 10, 5, and 3 kDa and revealed that peptides with MWs < 3 kDa had higher antioxidant activity than the other fractions.

These findings, however, do not imply that the smaller the MW, the better is the antioxidant activity. Jahanbani et al. [61] determined the antioxidant activities of <10, 5–10, 3–5, and <3 kDa fractions. Their results showed that the antioxidant activity of the peptide fractions was significantly lower than that of the whole hydrolysate, thus suggesting a synergistic effect between fractions with different MWs. Therefore, the appropriate regulation of MW is crucial to optimize the antioxidant activity of peptides.

### 4.2. Amino Acid Composition and Sequence

#### 4.2.1. Amino Acid Composition

The antioxidant activity of peptides is influenced by their amino acid composition. Previous studies have shown that the number and position of certain amino acids, such as hydrophobic amino acids (Leu, Val, Ala, Pro, and Phe), aromatic amino acids (Tyr, Trp, and Phe), sulfur amino acids (Cys and Met), acidic amino acids (Asp and Glu), and basic amino acids (His, Arg, and Lys), can contribute to the antioxidant activity of peptides [76]. Among these amino acids, the number and position of hydrophobic amino acids are particularly important as they can easily penetrate the lipid bilayer in the cell membrane and disrupt intracellular ROS levels. Moreover, the presence of hydrophobic amino acids may enhance the affinity and reactivity of peptides toward the cell membrane, thereby increasing the accessibility of the peptide to lipid-soluble ROS and contributing to the termination of lipid peroxidation [77,78].

Walnut proteins contain a high proportion of hydrophobic amino acids, which play a crucial role in the antioxidant activity of walnut peptides. Walnut peptides with high antioxidant activity have a higher proportion of hydrophobic amino acids than of hydrophilic amino acids. Therefore, the content of hydrophobic amino acids is one of the important indicators of the antioxidant activity of walnut peptides. It can promote the interaction between peptides and lipids and enhance the ability of peptides to inhibit lipid oxidation. Chen et al. [30] isolated a peptide (Ala–Asp–Ala–Phe) with three hydrophobic amino acids (75% of the peptide sequence) from walnuts; this might explain the peptide’s antioxidant activity in a linoleic acid model system.

However, excessive hydrophobic amino acids can reduce the solubility of the target peptide because of the influence of the carboxyl and amino groups on the side chain, thereby resulting in a lack of antioxidant properties. Hence, hydrophilic amino acids are equally important in the chelating activity of metal ions and the removal of HO· [79]. Acidic amino acids (Asp and Glu) and basic amino acids such as Arg and Lys can use the carbon and amino groups on the side chain as chelating agents for metal ions, quench unpaired electrons and free radicals, and have strong metal chelation ability, which is an important factor for peptides to eliminate free radicals [80,81]. Among these residues, the His residue has a strong ability to chelate metal ions, eliminate hydroxyl free radicals, and inhibit lipid peroxidation; these features can enhance the antioxidant capacity of peptides. This is mainly because the imidazole group on the histidine side chain can participate in hydrogen and electron transfer [82]. Wang et al. [23] determined the total and free amino acid compositions of WPH to investigate the effect of amino acid composition on the antioxidant activity of WPH. Acidic and basic amino acids accounted for 6.11% and 31.20% of the free amino acids in WPH, respectively, thus indicating that WPH is an effective free radical scavenger.

Aromatic amino acids such as Trp, Tyr, and Phe possess hydrogen donating capacity and are considered to exhibit excellent radical scavenging and antioxidant effects. Aromatic amino acids in WPH account for 11.77% and 30.09% of total and free amino acids, respectively. This finding suggests that WPH may have strong antioxidant activity [23]. In the study on the antioxidant activity of walnut peptides, Chen et al. [17] isolated the peptide Ala–Asp–Ala–Phe and confirmed the presence of aromatic amino acids such as phenylalanine through in vitro antioxidant activity assays. These amino acids were demonstrated to enable direct electron transfer to ROS. Li et al. [83] investigated the effect of tryptophan (Trp) on the antioxidant activity of walnut-derived peptides. The peptides containing Trp exhibited a high potential for antioxidant activity, and an increase in the content of Trp significantly enhanced the antioxidant activity of Trp-containing peptides.

Sulfur-containing amino acids, including cysteine (Cys) and methionine (Met), play a crucial role in the antioxidant activity of peptides. Met can be oxidized to methionine sulfoxide, resulting in antioxidant activity, while Cys can provide hydrogen sulfur, which directly reacts with free radicals [84]. In a study by Huiping et al., two peptide fractions were purified from walnut proteins, and 14 Cys- or Tyr-rich peptides with strong antioxidant activity were identified [32]. Cys, as a key amino acid residue in the peptide sequence, has the potential to capture free radicals, thus remarkably affecting the antioxidant capacity of the peptides.

In a study by Fan et al. [15], leucine (a hydrophobic amino acid), phenylalanine (a hydrophobic and aromatic amino acid), and proline (a basic amino acid) accounted for a large proportion of the 10 walnut peptides. This study further demonstrated the high antioxidant activity of WPH and confirmed that a higher content of hydrophobic amino acids in WPH resulted in better antioxidant activity. Moreover, an increase in the content of histidine and sulfur-containing amino acids also promoted the antioxidant activity of walnut peptides.

#### 4.2.2. Amino Acid Sequence

Previous studies have shown that the peptide sequence plays a crucial role in the antioxidant capacity of the peptide, particularly for intercellular antioxidant activity [85]. In another study, eight peptides with antioxidant activity were screened from walnut peptides by using ab initio sequencing, and appropriate selection criteria were used in this study, which involved the selection of amino acids with high antioxidant effects as parameters. Peptides without any common antioxidant amino acids exhibited in vitro antioxidant capacity similar to that of GSH, thus suggesting a greater effect of peptide sequence on the antioxidant activity of peptides [8].

The antioxidant capacity of peptides is influenced by various factors, with the amino acid composition of the N-terminal and C-terminal regions being a crucial determinant. The presence of hydrophobic amino acids in these regions can enhance the antioxidant activity of peptides [86]. Wang et al. [23] isolated and purified 48 walnut peptides and then evaluated their antioxidant activity. Notably, walnut peptides containing hydrophobic amino acids in the N-terminal or C-terminal region, such as QGRPWG, PSRADIY, and AYNIPVNIAR, exhibited strong antioxidant activity, thus highlighting the importance of hydrophobic amino acids in enhancing the antioxidant activity of walnut peptides. Fan et al. [15] investigated the relationship between the structural characteristics of peptides derived from WPH and their antioxidant activity. Their findings showed that, except for HADMVFY, all the 10 identified antioxidant peptides contained hydrophobic amino acids at either the N-terminal or C-terminal region, thus indicating that the presence of hydrophobic amino acids in these regions is associated with higher antioxidant activity.

Previous studies, however, have largely relied on speculative approaches rather than experimental validation to assess the effect of peptide sequences on the antioxidant activity of walnut peptides. To address this issue, synthetic peptides can be designed in which a single amino acid is replaced at a specific site in the peptide sequence, thus enabling the role of each amino acid to be confirmed by comparing the antioxidant activity of the synthetic peptide with that of the original target peptide [87]. This approach can help elucidate the potential relationship between the sequence of walnut-derived peptides and their antioxidant activity.

### 4.3. Secondary Structure

Regarding the structure of functional peptides, this review explores the effects of various structural factors on the antioxidant activity of walnut peptides and the underlying mechanisms. Peptides are typically chiral molecules with asymmetric secondary structures. Previous research has established a close relationship between the antioxidant activity of peptides and their secondary structures.

Secondary structure refers to the repetitive arrangement of amino acid residues in a polypeptide, which is stabilized by hydrogen bonds and van der Waals forces. α-helix is the most prevalent type of protein secondary structure, followed by β-fold. α-helix is stabilized by hydrogen bonds within the same helical structure, while hydrogen bonds are formed between different layers in β-fold. Other secondary structures include random coil and β-turn. β-turn plays a critical role in connecting chains such as α-helix and β-fold [88] through a ring between two chains.

Several studies have investigated the relationship between the secondary structure of walnut-derived peptides and their antioxidant activity. Fan et al. [15] found that, with the increase in enzymatic digestion time, ordered structures such as α-helix and β-turn angle gradually changed to β-fold and random coil, respectively, leading to the active site of the peptide chain and thus increasing the antioxidant activity of the hydrolyzed products as compared with that of the original walnut protein. The study showed that the number of α-helices and β-turns decreased, while the number of β-folds and random coils increased after hydrolysis; this finding indicated that the protein structure changed from ordered to disordered, thereby exposing hydrophobic groups in the protein’s internal structure and enhancing its antioxidant capacity [70]. This finding suggests that the secondary structure of peptides is an essential determinant of their antioxidant activity.

The relationship between the secondary structure and the antioxidant activity of walnut-derived peptides has not been extensively investigated in most studies. Therefore, the use of the quantitative SAR approach to explore this link could be a valuable topic for future research, which could contribute to the industrial application of walnut peptides. Through an in-depth analysis of the impact of secondary structure on antioxidant activity, we can gain a more comprehensive understanding of the underlying mechanisms and develop more effective strategies to enhance the antioxidant properties of walnut-derived peptides.

## 5. Conclusions

This article provides a comprehensive review of the techniques involved in the isolation, purification, and identification of walnut-derived peptides. The enzymatic hydrolysis process for obtaining antioxidant peptides as well as various in vitro and in vivo assays used to measure the antioxidant activity of the obtained peptides are discussed. The article highlights key factors such as amino acid MW, composition, and sequence and the secondary structure of the peptides that affect their antioxidant capacity. Recent studies investigating the mechanism of the effects of antioxidant peptides on cellular pathways are also briefly outlined.

The quest for natural antioxidants to replace synthetic iso-oxides is a crucial topic in international research. Walnut-derived antioxidant peptides have a high nutritional value because of their antioxidant properties and have the potential to become a viable alternative to synthetic antioxidants. These peptides could be used to develop nutritional functional foods for human consumption. However, the production of antioxidant peptides has been limited to laboratory settings, with few industrial applications to date. Most studies have focused on the preparation, purification, and characterization of antioxidant peptides, with fewer research studies conducted on their mechanism of action on cellular pathways. The constitutive relationship demonstrates significant advantages in evaluating the antioxidant activity of walnut peptides, even though the study of these peptides is still in its early stages. With further advances in science and technology, experimental industrialization is expected to be achieved in the near future. Moreover, in-depth exploration of the SAR and mechanism of action of walnut-derived antioxidant peptides could establish a solid foundation for their industrial application.

## Figures and Tables

**Table 1:** List of the commonly used methods for identifying walnut antioxidant peptides.

Enzyme Name	In Vitro Test Index	Isolation, Purification, and Identification	Enzymatic Conditions	Peptide Amino Sequences	Reference
Neutral protease, pineapple protease, pepsin, alkaline protease, papain, pancreatic protease	ABTS+ scavenging activity assay; DPPH free radical scavenging activity; Superoxide radical scavenging activity	Ultrafiltration membrane PVDF flat microporous membrane (MWCO of 200 kDa)	Neutral protease: pH: 7.0. Temperature: 50 °C, substrate concentration: 1:30 Alkaline protease: pH: 8.0. Temperature: 50 °C, substrate concentration 2:30 Pepsin: pH: 2.0. Temperature: 37 °C, substrate concentration: 1:30 Trypsin. pH: 8.0; temperature: 50 °C Enzyme/substrate: 2:100 (*w*/*w*) Pineapple protease: pH: 7.0. Temperature: 50 °C, substrate concentration: 3:30 Papain: pH: 7.0. Temperature: 50 °C, substrate concentration: 2:30	Walnut peptide	[1,2]
Alkaline protease	ORAC DPPH scavenging activity assay	Ultrafiltration UPLC-MS/MS	Alkaline protease: 23,400 U/mg pH: 8.0, temperature: 55 °C	QGRPWG, PSRADIY, AYNIPVNIAR	[3]
Trypsin	DPPH scavenging activity assay Fe^2+^ chelating capacity ABTS+ scavenging activity assay ORAC	Gel filtration chromatography RP-HPLC UPLC-ESI-MS/MS	Trypsin: 4.6 × 106 U/g pH: 7.5, temperature: 37 °C Enzyme/substrate: 2:100 (*w*/*w*)	YS, YK, YT, EM, CA, GR, YA, YG, LPC, CHC, SEK, GHC, YSVH	[4]
Pepsin, pancreatic protease	ABTS+ free radical scavenging activity. Oxygen radical absorbance capacity (ORAC).	Ultrafiltration Gel filtration chromatography. RP-HPLC. UPLC-QTOF-MS	Pepsin/substrate: 1:10 (*w*/*w*) pH: 2.0; temperature: 37 °C Trypsin/substrate: 1:10 (*w*/*w*) pH: 7.4; temperature: 37 °C	TY, SGGY.	[5]
Neutral protease, flavor protease, pepsin, and alkaline protease	DPPH free radical scavenging activity; ABTS+ Free radical scavenging activity; Hydroxyl radical scavenging activity;	Gel filtration chromatography HPLC LC-MS/MS	Neutral protease: 100 U/mg pH: 7.0; temperature: 50 °C Alkaline protease: 200 U/mg pH: 9.0; temperature: 55 °C Flavor protease: 30 U/mg pH: 7.5; temperature: 45 °C Pepsin: 500 U/mg pH: 2.0; temperature: 37 °C Enzyme/protein: 1:50 (*w*/*w*)	HADMVFY, NHCQYYL, NLFHKRP.	[6]
Alkaline protease	ABTS+ free radical scavenging activity; DPPH free radical scavenging activity; Hydroxyl radical scavenging activity Fe^2+^ chelating activity.	Ultrafiltration. Ion exchange chromatography. Gel filtration chromatography. RP-HPLC ESI-MS/MS	Alkaline protease: 62,000 U/g pH: 10.0; temperature: 55 °C Enzyme/substrate: 1:20 (*w*/*w*)	LAYLQYTDFETR	[7]
Alkaline protease	DPPH free radical scavenging activity; Hydroxyl radical scavenging activity;	SDS-PAGE Ion exchange column LC-HG-AFS	Alkaline protease: 4000 U/g, pH: 10.0, temperature: 45 °C, time: 4 h	Walnut peptide	[8]
Neutral protease, alkaline protease	DPPH free radical scavenging activity; Hydroxyl radical scavenging activity; Fe^2+^ chelating capacity	Ultrafiltration Gel filtration chromatography RP-HPLC ESI-MS/MS	Neutral protease: pH: 7.0, temperature: 50 °C Alkaline protease: pH: 8.0, temperature: 50 °C Pepsin: pH: 2.0; temperature: 37 °C	ADAF.	[9]
Alkaline protease	DPPH free radical scavenging activity; ORAC	Ion exchange column UPLC-MS/MS	Alkaline protease: 23,400 U/mg pH: 8.0, temperature:55 °C, time: 8 h	QGRPWG, GGEPRN, PSRADIY, AYNIPVNIAR,	[10]
Trypsin	Hydroxyl radical scavenging activity; ORAC	SP-825 macroporous absorption resin Medium-pressure liquid chromatography UPLC-ESI-MS/MS	Trypsin: 4.6 × 106 U/g pH: 8.0; temperature: 55 °C Enzyme/substrate: 1:20 (*w*/*w*)	WSREEQEREE, ADIYTEEAGR.	[11]
